# Supplementary material for: The impact of language on the interpretation of resuscitation clinical care plans by doctors. A mixed methods study
Source: PLoS One. 2019 Nov 25;14(11):e0225338. doi: 10.1371/journal.pone.0225338 (PMC6876871; doi:10.1371/journal.pone.0225338)
Supplement: S1 Fig — (DOCX) [file pone.0225338.s001.docx]

**Supplementary Information**

**S1 Fig: Copy of Resuscitation Plan-7 Step Pathwa**y


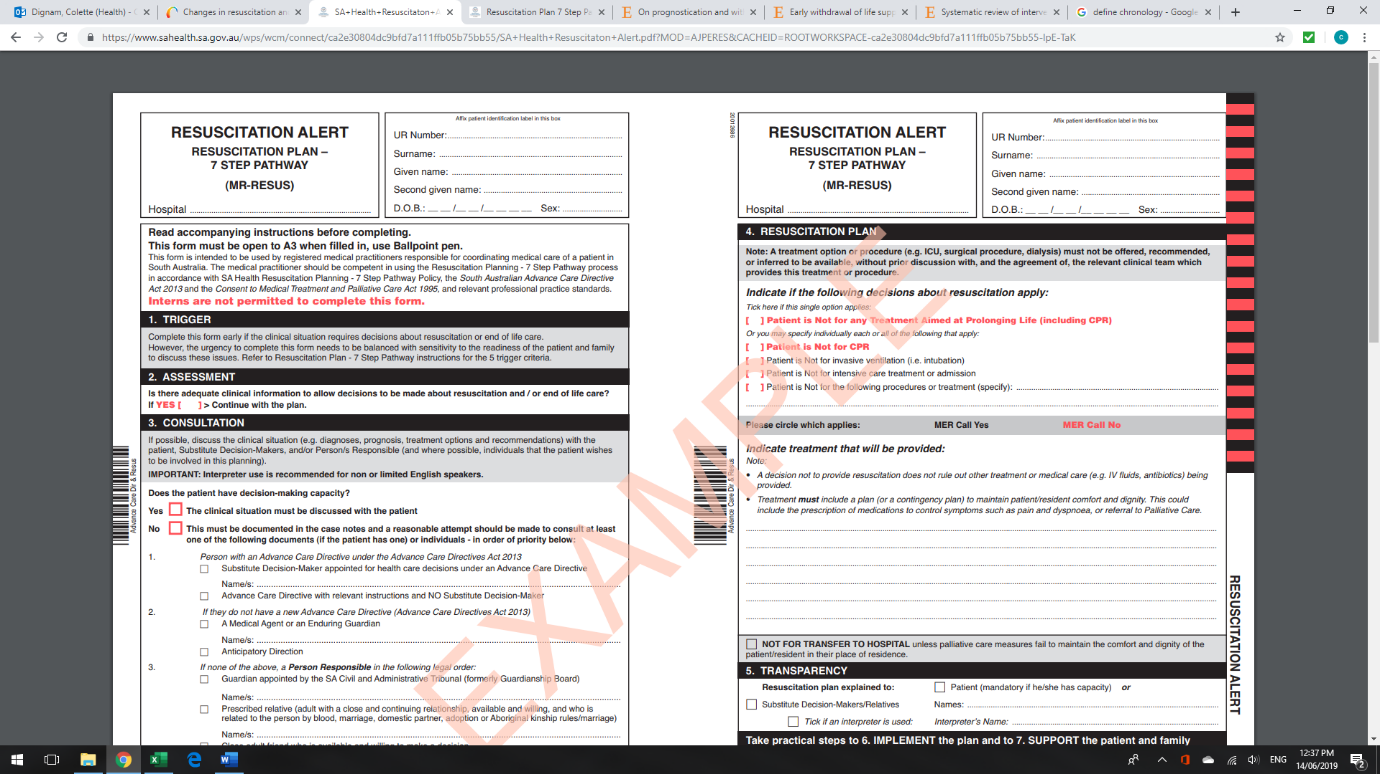

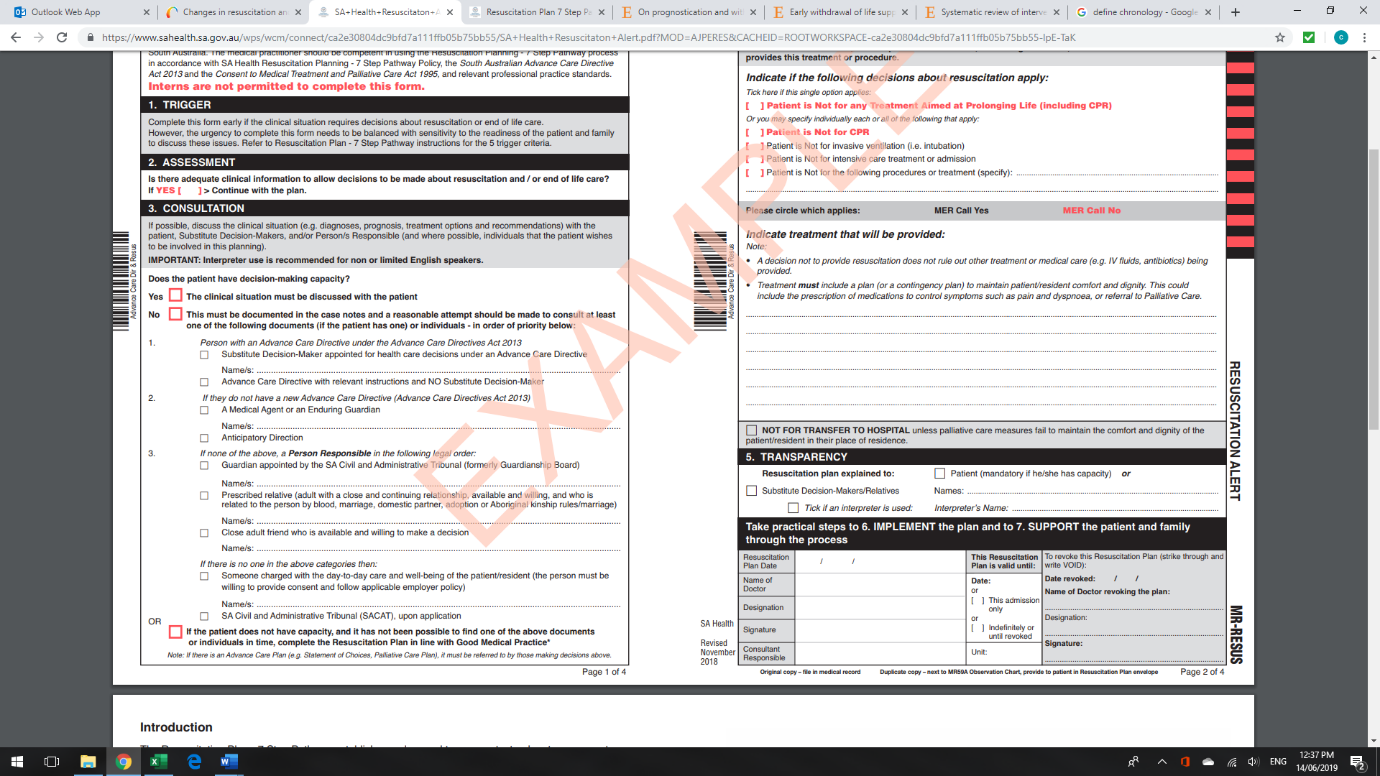


Available from:

www.sahealth.sa.gov.au/wps/wcm/connect/ca2e30804dc9bfd7a111ffb05b75bb55/SA+Health+Resuscitaton+Alert.pdf?MOD=AJPERES&CACHEID=ROOTWORKSPACE-ca2e30804dc9bfd7a111ffb05b75bb55-lpE-TaK
