# Supplementary material for: The impact of language on the interpretation of resuscitation clinical care plans by doctors. A mixed methods study
Source: PLoS One. 2019 Nov 25;14(11):e0225338. doi: 10.1371/journal.pone.0225338 (PMC6876871; doi:10.1371/journal.pone.0225338)
Supplement: S1 Text — (DOCX) [file pone.0225338.s002.docx]

**Supplementary Information**

**S1 Text: Unabridged Case Vignettes**

**Case #1**

# You review a patient for dyspnoea. He is admitted with end-stage COPD and severe pneumonia. Antibiotics were ceased yesterday. The 7-Step Pathway Resuscitation Plan states:

# Patient is Not for any treatment aimed at Prolonging Life (including CPR).

# In the free text box indicating treatment that will be provided: Comfort care.

# Which of the following measure(s) would you consider appropriate in this man?

**Case #2**

A 78-year old patient from high level of care nursing home is admitted with urosepsis. She is agitated. She has a history of advanced dementia and is non-verbal. She is 2-assist and receives a pureed diet. Her Advance Care Directive states in the binding refusals section; 'If my dementia gets worse and I cannot make my own decisions, I do not want any treatment aimed at sustaining life.' There is no contactable Substitute Decision Maker. A 7-Step Pathway Resuscitation Plan was completed during a prior admission and remains active. It states:

Not for any Treatment aimed at Prolonging Life (including CPR).

Not for MET Calls.

There is no further detail.

Which of the following treatments would you deem suitable to instigate in this patient?

**Case #3**

A 70-year old patient has a MER call for hypotension and bradycardia. She was admitted after being found unconscious at home. She is very drowsy and unable to engage in conversation. She has a history of atrial fibrillation and metastatic breast cancer in 2015, which was reportedly cured. She is prescribed metoprolol 50mg BD. There is no appointed Substitute Decision Maker or Advance Care Directive. A-Patent B- RR 18. Sats 97%RA. Chest clear. C- 90/40. HR 40. Reduced urine output. D-GCS 14. BGL 7.6. ECG shows junctional bradycardia with a rate of 30bpm.There is no evidence of sepsis. The patient’s 7-Step Pathway Resuscitation Plan was completed by the admitting doctor following discussion with her husband. It states:

Patient is Not for CPR

Patient is Not for invasive ventilation (ie intubation)

Patient is Not for intensive care treatment or admission

MER Call: YES

In the free text box indicating treatment that will be provided: Active ward measures

Which of the following management would you instigate at this MER call (circle all those you would be willing to instigate given limits of care outlined in the 7-Step Pathway Resuscitation Plan?
