# Supplementary material for: The impact of language on the interpretation of resuscitation clinical care plans by doctors. A mixed methods study
Source: PLoS One. 2019 Nov 25;14(11):e0225338. doi: 10.1371/journal.pone.0225338 (PMC6876871; doi:10.1371/journal.pone.0225338)
Supplement: Attachment — Submitted filename: PONE-D-19-20806_reviewer.docx [file pone.0225338.s003.docx]

PLOS ONE

#### De-coding the language; how clinicians interpret resuscitation clinical care plans. A mixed methods study

--Manuscript Draft--

| Manuscript Number: | PONE-D-19-20806 |
| --- | --- |
| Article Type: | Research Article |
| Full Title: | De-coding the language; how clinicians interpret resuscitation clinical care plans. A mixed methods study |
| Short Title: | De-coding the language; how clinicians interpret resuscitation clinical care plans |
| Corresponding Author: | Colette Dignam  The University of Adelaide  Adelaide, South Australia AUSTRALIA |
| Keywords: | resuscitation documentation; goals of care; end of life; CPR; NFR; Language |
| Abstract: | Introduction  Resuscitation clinical care plans (resuscitation plans) are gradually replacing ‘Not for Cardiopulmonary Resuscitation’ orders in the hospital setting. The 7-Step Pathway Resuscitation Plan and Alert form (7-Step form) is one example of a resuscitation plan. Treatment recommendations in resuscitation plans currently lack standardised language, creating potential for misinterpretation and patient harm.  Aims  To explore how terminology used in resuscitation plans is interpreted and applied by clinicians.  Method  A mixed methods study surveyed 50 general medical doctors, who were required to interpret and apply a 7-Step form in three case vignettes and define seven key terms. Statistical analysis on multiple choice and thematic analysis on free-text responses was performed.  Results  Terminology was neither consistently interpreted nor applied, resulting in clinically significant differences in treatment choices. Three key themes influenced the application of a resuscitation plan: in-depth discussion, precise documentation and personal experience of the bedside deciding doctor.  Discussion  This study highlights persistent communication deficiencies in resuscitation plan documentation and how this may adversely affect patient care; findings unlikely to be unique to Australia or South Australia.  Conclusion  Removing ambiguity by standardising and defining the terminology in resuscitation plans will improve bedside decision-making, while also supporting the rights of the patient to receive appropriate and desired care. |
| Order of Authors: | Colette Dignam |
|  | Josephine Thomas |
|  | Margaret Brown |
|  | Campbell H Thompson |

| Additional Information: |  |
| --- | --- |
| Question | Response |
| Financial Disclosure  Enter a financial disclosure statement that describes the sources of funding for the work included in this submission. Review the [submission guidelines](http://journals.plos.org/plosone/s/submission-guidelines#loc-financial-disclosure-statement) for detailed requirements. View published research articles from [PLOS ONE](http://journals.plos.org/plosone/) for specific examples.  This statement is required for submission and will appear in the published article if the submission is accepted. Please make sure it is accurate. | The authors received no specific funding for this work. |
| Unfunded studies  Enter: The author(s) received no specific funding for this work.  Funded studies  Enter a statement with the following details:   - Initials of the authors who received each award - Grant numbers awarded to each author - The full name of each funder - URL of each funder website - Did the sponsors or funders play any role in the study design, data collection and analysis, decision to publish, or preparation of the manuscript? - NO - Include this sentence at the end of your statement: The funders had no role in study design, data collection and analysis, decision to publish, or preparation of the manuscript. - YES - Specify the role(s) played. |  |
| * typeset |  |
| Competing Interests  Use the instructions below to enter a competing interest statement for this submission. On behalf of all authors, disclose any [competing interests](http://journals.plos.org/plosone/s/competing-interests) that could be perceived to bias this work—acknowledging all financial support and any other relevant financial or non- financial competing interests. | The authors have declared that no competing interests exist. |

| This statement will appear in the published article if the submission is accepted. Please make sure it is accurate. View published research articles from [PLOS ONE](http://journals.plos.org/plosone/) for specific examples. |  |
| --- | --- |
| NO authors have competing interests  Enter: The authors have declared that no competing interests exist.  Authors with competing interests  Enter competing interest details beginning with this statement:  I have read the journal's policy and the authors of this manuscript have the following competing interests: [insert competing interests here] |  |
| * typeset |  |
| Ethics Statement  Enter an ethics statement for this submission. This statement is required if the study involved:   - Human participants - Human specimens or tissue - Vertebrate animals or cephalopods - Vertebrate embryos or tissues - Field research   Write "N/A" if the submission does not require an ethics statement.  General guidance is provided below. Consult the [submission guidelines](http://journals.plos.org/plosone/s/submission-guidelines#loc-guidelines-for-specific-study-types) for detailed instructions. Make sure that all information entered here is included in the Methods section of the manuscript. | Written ethics approval was obtained through the Royal Adelaide Hospital Ethics and Governance committee: HREC/17/RAH/375 |

| Format for specific study types  Human Subject Research (involving human participants and/or tissue)   - Give the name of the institutional review board or ethics committee that approved the study - Include the approval number and/or a statement indicating approval of this research - Indicate the form of consent obtained (written/oral) or the reason that consent was not obtained (e.g. the data were analyzed anonymously)   Animal Research (involving vertebrate animals, embryos or tissues)   - Provide the name of the Institutional Animal Care and Use Committee (IACUC) or other relevant ethics board that reviewed the study protocol, and indicate whether they approved this research or granted a formal waiver of ethical approval - Include an approval number if one was obtained - If the study involved non-human primates, add additional details about animal welfare and steps taken to ameliorate suffering - If anesthesia, euthanasia, or any kind of animal sacrifice is part of the study, include briefly which substances and/or methods were applied   Field Research  Include the following details if this study involves the collection of plant, animal, or other materials from a natural setting:   - Field permit number - Name of the institution or relevant body that granted permission |  |
| --- | --- |
|  |  |
| Data Availability  Authors are required to make all data underlying the findings described fully available, without restriction, and from the time of publication. PLOS allows rare exceptions to address legal and ethical concerns. See the [PLOS Data Policy](http://journals.plos.org/plosone/s/data-availability) and [FAQ](http://journals.plos.org/plosone/s/data-availability#loc-faqs-for-data-policy) for detailed information. | No - some restrictions will apply |

| A Data Availability Statement describing where the data can be found is required at submission. Your answers to this question constitute the Data Availability Statement and will be published in the article, if accepted. |  |
| --- | --- |
| Important: Stating ‘data available on request from the author’ is not sufficient. If your data are only available upon request, select ‘No’ for the first question and explain your exceptional situation in the text box. |  |
| Do the authors confirm that all data underlying the findings described in their manuscript are fully available without restriction? |  |
| Describe where the data may be found in full sentences. If you are copying our sample text, replace any instances of XXX with the appropriate details.   - If the data are held or will be held in a public repository, include URLs, accession numbers or DOIs. If this information will only be available after acceptance, indicate this by ticking the box below. For example: All XXX files are available from the XXX database (accession number(s) XXX, XXX.). - If the data are all contained within the manuscript and/or Supporting Information files, enter the following: All relevant data are within the manuscript and its Supporting Information files. - If neither of these applies but you are able to provide details of access elsewhere, with or without limitations, please do so. For example:   Data cannot be shared publicly because of [XXX]. Data are available from the XXX Institutional Data Access / Ethics Committee (contact via XXX) for researchers who meet the criteria for access to confidential data.  The data underlying the results presented in the study are available from (include the name of the third party | Data cannot be shared publicly because permission was not obtained from survey participants to share their answers in a public database. This is in accordance with the current ethics approval. Data are available from the corresponding author once permission is obtained from the Royal Adelaide Hospital ethics committee (contact via health.CALHNresearchLNR@sa.gov.au) for researchers who meet the criteria for access to confidential data. |

| and contact information or URL).   - This text is appropriate if the data are owned by a third party and authors do not have permission to share the data.   * typeset |  |
| --- | --- |
| Additional data availability information: |  |

Manuscript	[Click here to download Manuscript Manuscript PLOS.docx](https://www.editorialmanager.com/pone/download.aspx?id=24868911&amp;guid=c288168c-ffb4-4239-90ba-c68f9864c773&amp;scheme=1)

1

2

3

4

5

## 6 De-coding the language; how clinicians interpret resuscitation

7 **clinical care plans. A mixed methods study.**

8

9

10

11	Colette Dignam^1,2*^, Josephine Thomas 3¶, Margaret Brown 4¶, Campbell H Thompson 1,2¶

12

13

14

15

16

17

18 1 Division of Medicine, Faculty of Health and Medical Sciences, University of Adelaide,
19 Adelaide, South Australia, Australia 20

21	2 Division of Medicine, Royal Adelaide Hospital, Adelaide, South Australia, Australia 22

23 3 Adelaide Medical School, Faculty of Health and Medical Sciences, University of Adelaide,
24 Adelaide, South Australia, Australia 25
26 4 School of Psychology, Social Work, and Social Policy. Division of Education, Arts and
27 Social Sciences. University of South Australia, Adelaide, South Australia, Australia 28

29

30

31

32 ***** Corresponding author:
33 Ema[il: colette.dignam@sa.gov.au](mailto:colette.dignam@sa.gov.au) (CD) 34

35

36	¶ These authors contributed equally to this work. 37

38

39

40

41	**Abstract word count:** 206 42

43	**Manuscript word count:** 2885 44

# 45 Abstract:

46 **Introduction:** Resuscitation clinical care plans (resuscitation plans) are gradually replacing
47 ‘Not for Cardiopulmonary Resuscitation’ orders in the hospital setting. The 7-Step Pathway
48 Resuscitation Plan and Alert form (7-Step form) is one example of a resuscitation plan.
49 Treatment recommendations in resuscitation plans currently lack standardised
50 language, creating potential for misinterpretation and patient harm.
51 **Aims:** To explore how terminology used in resuscitation plans is interpreted and applied by
52 clinicians.
53 **Method:** A mixed methods study surveyed 50 general medical doctors, who were required to
54 interpret and apply a 7-Step form in three case vignettes and define seven key terms.
55 Statistical analysis on multiple choice and thematic analysis on free-text responses was
56 performed.
57 **Results:** Terminology was neither consistently interpreted nor applied, resulting in clinically
58 significant differences in treatment choices. Three key themes influenced the application of a
59 resuscitation plan: in-depth discussion, precise documentation and personal experience of the
60 bedside deciding doctor.
61 **Discussion:** This study highlights persistent communication deficiencies in resuscitation plan
62 documentation and how this may adversely affect patient care; findings unlikely to be unique
63 to Australia or South Australia.
64 **Conclusion:** Removing ambiguity by standardising and defining the terminology in
65 resuscitation plans will improve bedside decision-making, while also supporting the rights of
66 the patient to receive appropriate and desired care.

67

# 68 Introduction:

69 A resuscitation clinical care plan (resuscitation plan) is a document written by a clinician
70 responsible for a patient’s care which includes a directive regarding cardiopulmonary
71 resuscitation (CPR), but also contains recommendations and/or limitations about other
72 interventions. Resuscitation plans encourage discussions about broader goals of care, with
73 an informed medical assessment of likely treatment outcomes and the patient/substitute
74 decision maker’s (SDM) treatment preferences taken into account [1,2].

75

76 Following its launch in 2014, the 7-Step Pathway Resuscitation Plan and Alert form (7-Step
77 form) (S1 Fig 1) is now the resuscitation plan used in all South Australian public tertiary
78 hospitals [3]. The form is supported by government policy promoting transparent
79 communication, patient-centred decision-making, and the right to withhold futile
80 interventions [3,4]. Like international counterparts, the document includes a CPR directive,
81 treatment limitations that can be selected using tick-boxes, and a section to write further
82 details about appropriate care [1,5,6].

83

84 Previous analysis has shown that when compared to ad hoc case-note documentation, the 7-
85 Step form improved frequency of resuscitation documentation, but did not reduce the use of
86 undefined and ambiguous terminology [4]. International resuscitation plans are similarly
87 replete with ambiguous or undefined terms including ‘ward care’, ‘supportive care’ and
88 ‘limited intervention’[7-9]. The lack of standardised language may create a risk of
89 inappropriate patient care through misinterpretation.

90

91 This study investigates whether terminology used in a resuscitation plan is consistently
92 interpreted and applied by a clinician at the bedside. Secondary aims were to determine
93 whether clinicians found a standardised resuscitation plan useful, and to explore the other
94 factors which influence how they applied a resuscitation plan.

95

96

# 97 Method:

98 A mixed methods approach was adopted to maximise insight into resuscitation plan
99 interpretation. An electronic survey was distributed to all doctors who worked in General
100 Medicine at a single tertiary hospital between February-August 2017. The 100 eligible
101 doctors received an email invitation, and two follow-up emails. Participation was anonymous,
102 with demographic data limited to education about resuscitation documentation and job

seniority.

105 In the first section of the survey, three case vignettes drawn from real life examples were
106 described (S2- Unabridged Case Vignettes). Having read the resuscitation plan, participants
107 selected all interventions they deemed appropriate from a list of multiple-choice options. In
108 the second section, participants defined seven terms used in the free-text section of the 7-Step
109 form; identified from previous research [4]. The final section was a Likert Scale response to
110 the statement ‘the 7-Step Pathway aids decision-making about appropriate goals of care, not
111 just CPR’, with options ranging from strongly agree to strongly disagree. Participants could

expand on all responses with free-text.

114 The Fisher-exact test was used to compare the treatment choices of senior and junior doctors
115 and differences were highlighted where statistical significance was reached (p=0.05). In each
116 case vignette, responses were also ranked from most consistently chosen to least consistently
117 chosen, with 100% or 0% representing maximum agreement between respondents,
118 and 50% representing maximum disagreement. The ranked responses were used to calculate a standard
119 deviation that quantified the relative level of agreement between respondents’ answers in
120 each case. Standard deviations of the three case vignettes were compared using Lehmans’
121 variance ratio to determine statistical significance (p=0.05).
122 Thematic analysis was performed on the free-text and semi-structured responses using the
123 established methodology of Braun and Clark [10]. Each respondent was assigned a unique
124 identifier including a number and a letter (C=Consultant, R=Registrar, M=Resident Medical
125 Officer, I=Intern). Following data familiarisation, preliminary concepts from each
126 participant’s responses were identified and inductively coded. Themes and subthemes were
127 sought until all data was reviewed or saturation reached. The second author and a local expert
128 with experience in both the subject matter and qualitative research independently reviewed
129 the data to ensure all themes were adequately captured and no themes or divergent views
130 were missed. Links between free-text responses and multiple-choice treatment selections
131 were sought and new themes developed or incorporated into existing themes where
132 appropriate. Ethics approval was obtained: HREC/17/RAH/375.

# 134 Results:

### 135 Characteristics of participants

136 50 responses were obtained, predominantly from interns and residents (Table 1). This is
137 consistent with the General Medicine ward structure where the junior staff rotate every three
138 months and therefore total numbers are larger. 31/50 (62%) participants had completed
139 education about the 7-Step form, chiefly via attendance at in-hospital presentations. There
140 was no statistical difference in the prevalence of those who received education across the

levels of seniority.

147 **Table 1: Characteristics of participants**

|  | Intern | Resident | Registrar | Consultant | TOTAL |
| --- | --- | --- | --- | --- | --- |
| Participants (%  of invitees) | 23 (48) | 17 (52) | 3 (38) | 7 (37) | 50 (50) |
| Had received  education (%) | 13/23 (57) | 11/17 (65) | 2/3 (67) | 5/7 (71) | 31/50 (62) |
| Agreed* (%) | 20/23 (87) | 13/17(76) | 2/3 (67) | 4/7 (57) | 39/50 (78) |

148 *These doctors agreed or strongly agreed with the statement ‘The 7-Step Pathway aids decision-making about
149 appropriate goals of care, not just CPR’
151 Overall, 39/50 (78%) of doctors agreed or strongly agreed that the 7-Step form aids decision-
152 making about appropriate goals of care, not just CPR. There was no association between
153 education received and the clinicians’ opinion of its usefulness (p=0.9). 87% of interns
154 agreed the 7-Step form is a useful decision aid, compared to 57% of consultants. The
155 difference did not reach statistical significance (p=0.08).

### 157 Case vignette interpretation

158 (See S2 for Unabridged case vignettes)
159 ***Case #1-Comfort Care:*** *A dyspnoeic patient with End-Stage Chronic Obstructive Pulmonary*
160 *Disease has deteriorated despite maximum therapy. The 7-Step form tick-box section states*

*‘Not for any Treatment Aimed at Prolonging Life’. The free-text states ‘Comfort care’.*

163 ***Case #2 -No Life Prolonging Treatment:*** *A patient with end-stage dementia presents with*
164 *likely urosepsis. The patient’s Advance Care Directive states ‘If my dementia gets worse and*
165 *I can no longer make my own decisions, I do not want any treatment to sustain life.’ The 7-*
166 *Step form tick-box section states ‘Not for any Treatment Aimed at Prolonging Life/Not for*
167 *MER (Medical Emergency Response) calls’. The free-text is blank.*
168 ***Case #3- Ward Measures:*** *A patient is reviewed at a MER call for profound drowsiness,*
169 *hypotension and heart rate 30bpm. She has a history of atrial fibrillation on metoprolol 50mg*
170 *twice daily, and quiescent metastatic breast cancer. A 7-Step form was previously completed*
171 *with her husband. The tick-box section states ‘Not For CPR/invasive ventilation/intensive*

*care treatment or admission’. The free-text states ‘Active ward measures’.*

174 The treatment choices selected in response to each case vignette are shown in Fig 1A, 1B and
175 1C. In Case #1-Comfort Care, 96% of respondents selected subcutaneous morphine and did
176 not select intravenous fluid bolus, chest X-ray or arterial blood gas. Consultants were more
177 likely than junior doctors to select intravenous antibiotics, chest x-ray and arterial blood gas
178 (p=0.04 in all three examples). Supplemental oxygen using nasal specs and non-rebreather
179 were selected by 72% and 18% respectively. It was unclear from free-text responses if

oxygen was given for symptomatic relief or to extend life.

**Fig 1A: Case #1- Comfort care: Percentage of respondents who agreed with each treatment option**

^+^Where a treatment was selected by <50%, the inverse proportion has been displayed in order to demonstrate degree of concordance between doctors ie 6% selected antibiotics so 94% selected (No) antibiotics. ^Non-rebreather. *Intravenous.

**Fig 1B: Case #2- No life prolonging treatment: Percentage of respondents who agreed with each treatment option**

^+^As in Fig 1A, the inverse proportion has been graphically displayed. ^Microscopy, sensitivities and culture. *In-dwelling urinary catheter. ~Computerised Tomography scan

**Fig 1C: Case #3- Ward measures: Percentage of respondents who agreed with each treatment option**

^+^As in Graph 1, the inverse proportion has been graphically displayed. ^Coronary Care Unit

193 In Case#2-No Life Prolonging Treatment, morphine and midazolam were selected by 82%
194 and 85% of responders respectively**.** Potentially life-prolonging options were selected by 26%
195 of respondents, including intravenous fluid (26%), intravenous antibiotics (24%) and oral
196 antibiotics (14%). 42% of consultants prescribed intravenous antibiotics versus 23% of junior

staff, though the difference was not statistically significance (p=0.3).

199 In Case#3-Ward Measures, more than 90% of respondents selected the options of intravenous
200 fluid bolus, electrolyte replacement and cardiology consult. Intravenous atropine, coronary
201 care unit admission and external cardiac pacing were selected by 78%, 66% and 50% of
202 respondents respectively. Consultants were more likely to give isoprenaline and external

pacing than junior doctors (p=0.03).

205 The quantified level of agreement between clinicians about appropriate treatment was
206 greatest in Case#1, then Case #2, then Case #3 with standard deviations of 10.8, 11.7 and
207 19.3 respectively. The difference in level of agreement between Case #1 and Case #3 was
208 statistically significant (p<0.001), suggesting that ‘comfort care’ results in a more consistent
209 approach to management than ‘active ward measures’.

### 211 Definitions of key terms in resuscitation plans

212 48 doctors completed this section. 29 (60%) participants used one repeated definition for all
213 five ‘ward-’ terms or wrote ‘*as above’*, indicating they deemed these terms synonymous. For
214 respondents who gave multiple definitions, the intent of treatment and the perceived
215 appropriate limit of care varied significantly, as seen in Table 3. ‘Active measures’ and ‘full
216 ward measures’ were associated with the provision of more aggressive treatments, including

CPR for 20% of respondents.

225 **Table 3: Selected definitions of key terms**

| **Key term to define** | **Selected definitions** |
| --- | --- |
| Full ward measures | *“Everything possible, including resuscitation”* (M4)  *“Everything except ICU/CPR/intubation”* (M6) |
| Active ward measures | *“I find this unclear-unsure if it would include escalation to ICU”* (M2) |
| Ward based measures | *“An intervention which can be safely performed on a ward; not necessarily to*  *prolong life”* (I21) |
| Ward based management | *“Treatment outside of ICU, aim to cure/prolong life. May include a procedure*  *depending on the situation.”* (C2) |
| Ward based care | *“Measures which can be given on a ward but which do not actively pursue a new*  *diagnosis”* (I10) |
| Comfort Care | *“Aimed at allowing the patient to die with dignity with relief from symptoms such*  *as pain, dyspnoea, agitation.”* (I9) |
| Palliative Approach | *“This is a very ambiguous term, palliative approach can mean ensuring best quality of life…thus may also involve some active management” (C4)*  *“Comfort care for patient certain to die” (C5)* |

227 For ‘comfort care’ and ‘palliative approach’, goals of care were reflected in language like
228 ‘s*ymptomatic relief*’, ‘*dying’* and ‘*alleviate distress*’, with 31/47 (65%) of respondents using a
229 single definition for both terms. In responses where the terms were separately defined, the
230 chronology between ‘palliative approach’, ‘comfort care’ and imminent death varied.
231 Opinions varied about whether artificial hydration, antibiotics and chemotherapy were part of
232 a ‘palliative approach’, especially in patients presenting with an illness other than their
233 underlying life-limiting condition. Of the seven terms, ‘comfort care’ was the term where

intent of care was the most concordant.

### 238 Thematic analysis:

239 Analysis of free-text responses revealed three key themes that influenced how a resuscitation
240 plan is interpreted and applied. These were ‘the discussion’, ‘the documentation’ and ‘the

deciding doctor’.

243 The theme, ‘the discussion’, pertains to the communication between the documenting clinician
244 and patient/SDM prior to completion of a resuscitation plan. A robust and clearly
245 documented discussion was valued by respondents, and considered pivotal to implementing
246 the clinical care plan. When the 7-Step form was incomplete or there was inadequate
247 supporting documentation describing the discussion, respondents acknowledged

misinterpretation was possible.

250 In Case#3-Ward Measures, participants perceived that inadequate discussion had occurred
251 and expressed reduced confidence in the documented resuscitation plan. They indicated a
252 reduced willingness to make clinical decisions without additional information. Participants
253 suggested that when the initial discussion was not sufficiently detailed, workload and patient
254 care were both adversely affected. A pre-emptive or early discussion was preferred to

impromptu discussion prompted by patient deterioration.

257 *“I have been [in] situations before where 7-Steps have been misinterpreted as that relevant*
258 *box has not been discussed with the patient.”* (I6)
259 *“Every day multiple METs occur where someone in T2RF [Type 2 Respiratory Failure] is*
260 *"Not for CPR/ICU/[intubation]” but there is no mention of NIV [Non-invasive ventilation] …*

*resulting in multiple phone calls to determine the true ceiling of care”* (M15)

263 The theme, ‘the documentation’, encompasses the available written information at the
264 bedside in both the 7-Step form and/or case notes. The change to a standardised resuscitation
265 plan was viewed positively because of ease of locating and increased clarity, though not all

respondents felt it had changed their practice.

268 The form was reported to clearly indicate if a patient should not receive CPR, and when the
269 focus was strictly palliative. However, respondents were critical of how the free-text section
270 was used; ill-defined or general statements were reportedly common and rarely added
271 clarification to treatment goals. In particular, the phrase ‘ward measures’ was perceived as a
272 source of frustration and ambiguity. Since treatments available in each ward vary, it was
273 unclear to the respondents what treatments were actually appropriate. The addition of other
274 specific treatments (e.g. non-invasive ventilation) to the tick-box section was suggested to

clarify this uncertainty.

277 *“The 7-step pathway has not changed how I document my resuscitation plans or how I have*
278 *the discussion with patients and families. It has provided a common place to write the*
279 *resuscitation orders in the case notes.”* (R2)
280 *“There should be also a move to further specify treatments that are*
281 *appropriate/inappropriate as many 7-steps end up with very vague and generalised*
282 *statements.”* (M14)
283 *"Ward based measures" is okay, but fails to adequately identify the ceiling limit of care. Lots*

*of therapies can take place on the wards, especially on "specialty wards*." (M15)

286 The theme, ‘the deciding doctor’, acknowledges the bedside clinician as an individual, whose
287 treatment decisions are affected by personal values and experience. Interns were unlikely to
288 question the resuscitation plan’s instructions and valued specific limitations that could be
289 readily followed. For more senior doctors, there were conflicting views of the resuscitation
290 plan’s role in determining care, ranging from a useful tool to a guideline that stood in the way

of individualised patient assessment.

293 The deciding doctors’ treatment choices were therefore impacted by the value they placed on
294 the resuscitation plan and its specified treatment limitations, with some doctors willing to
295 overrule the plan (or at least re-open the discussion). This concept was evident in the free text

responses and the treatment selections in case vignettes.

298 *“[The] decision has to be made on individual circumstances on the day, protocols can be a*
299 *help or a distraction.”* (C5)
300 *“As with anything, the more information provided by the person who is writing the 7-step,*
301 *the more useful it is to determine the appropriate goals of care.”* (I21)
302 Re Case #3: *“In this case I'd probably ignore the 7-steps if what was discussed wasn't*
303 *documented, and I'd call the patients (sic) consultant too.”* (M8) 304

# 305 Discussion:

306 Despite resuscitation plans being regarded as a useful tool by 78% of respondents, this study
307 shows that much of the terminology used in the 7-Step form is inconsistently interpreted and

applied by doctors, resulting in clinically significant differences in treatment choices.

310 Responses to all of the case vignettes showed treatment inconsistencies, with Case#3-Ward
311 Measures showing least agreement about appropriate management. In Case#1-Comfort Care,
312 there was inconsistent provision of oxygen and fluids by respondents despite definitions of
313 ‘comfort care’ suggesting a (lack of?) mutually shared understanding of treatment intent. This finding is
314 similar to a USA study where 176 surveyed clinicians had disparate opinions about whether
315 ‘comfort measures only’ included intravenous antibiotics, supplemental oxygen or emergency

response calls [10].

318 In Case #2-No Life Prolonging Treatment, 24% of respondents prescribed potentially life-
319 saving intravenous antibiotics for a septic patient whose binding Advance Care Directive
320 stated ‘No treatment to sustain life’. Prescribing antibiotics is at best a problematic
321 misinterpretation, making clear the need for standardised definitions. It could also be a
322 paternalistic disregard for a patient’s right to refuse medical treatment. This is not an isolated
323 occurrence; one USA study found 2-13% of surveyed doctors would provide CPR for

patients with a ‘Do Not Resuscitate’ order [12].

326 In Case #3-Ward measures, uncertainty was greatest about whether sub-specialty emergency
327 interventions like isoprenaline infusion and external pacing were appropriate. Uncertainty
328 was also evident in the range of definitions given for each of the five ‘ward-’ terms: while
329 60% of respondents would assume the same goals of care regardless of whether ‘full ward
330 measures’ or ‘ward based care’ was documented, the remaining 40% of doctors thought each
331 term reflected a different treatment approach, including whether they should aim to prolong

life.

334 The implementation of a resuscitation plan is confounded by factors other than ambiguous
335 language. Confidence in a resuscitation plan was greatest when there was a robust and clearly
336 documented discussion with adequately detailed treatment limitations. Even when adequately
337 detailed, the bedside deciding doctor may re-discuss, ignore, or flexibly interpret a
338 resuscitation plan they disagreed with. Interns, who are more reliant on senior direction, were
339 more likely to both closely follow a resuscitation plan and consider it a useful decision-
340 making tool. International studies have also shown that resuscitation plans are relied upon by
341 after-hours junior doctors to accurately convey the home team’s treatment recommendations,
342 and improve speed of decision-making [13,14]. To further improve clarity for junior staff,
343 free-text options could be replaced with standardised choices e.g. non-invasive ventilation,

cardiac monitoring, artificial hydration and intravenous antibiotics.

346 While not the focus of this study, the results suggest that the perceived purpose of a
347 resuscitation plan varies between doctors: is it predominantly a handover tool, or a document
348 to protect patients from unwanted intervention? These objectives should ideally align, but
349 translation of a patient’s wishes into documented treatment limitations is an imprecise
350 process. Misinterpreting a resuscitation plan based on an Advance Care Directive, or relying
351 on poor quality documentation, does not simply represent a failed medical handover. It
352 represents a loss of the patient’s voice in the decision-making process. The underlying aim of
353 resuscitation planning policy, to champion the patient’s voice, has arguably not been

achieved [15].

356 This study is limited to a single-centre general medical unit with 50% response rate which
357 may limit generalisability of findings. A strength of the selected cohort is that this general
358 medical unit completes resuscitation plan forms on 63% of patients over 70 years, and has
359 been using the 7-Step form in its current format since 2014. Sampling from this cohort
360 therefore, removes confounding factors such as form variability or human error due to
361 unfamiliarity with the form. The written format of the survey may have resulted in less
362 comprehensive responses than verbal interviews.

# 363 Conclusion:

364 Terminology in resuscitation plans is neither consistently interpreted nor applied, which
365 affects bedside treatment decisions. The deciding doctor is also influenced by personal
366 experience, seniority, and the perceived quality of discussions with the patient/SDM.
367 Ambiguous language in resuscitation plans undermines a key tenet of resuscitation
368 documentation, to record a patient’s treatment preferences in a manner that will be accurately
369 interpreted and faithfully upheld. Moving towards universally shared and defined
370 terminology in resuscitation plans will improve medical handover, increase physician
371 confidence in the value of the documentation and ultimately, support delivery of appropriate
372 and desired care for the patient.
374 **Acknowledgements:** The authors thank Paul Hakendorf for his statistical expertise 375

376

377

378

379

380

381

382

383

384

385

386

387

388

389

390

391	**References:**

392

393 1.	Fritz Z, Slowther AM, Perkins GD. Resuscitation policy should focus on the patient,
394 not the decision. BMJ. 2017;356:1-6.
395 2.	Citko J, Moss AH, Carley M, Tolle S. The National POLST Paradigm Initiative, 2nd 396	Edition #178. J Palliat Med. 2011;14:241-2.
397 3.	SA Health. Resuscitation Plan 7 Step Pathway for Health Professionals. Department
398 for Health and Ageing. South Australia: Government of South Australia; 2014. (Accessed 16
399 June 2019, at
400 [www.sahealth.sa.gov.au/wps/wcm/connect/public+content/sa+health+internet/clinical+resour](http://www.sahealth.sa.gov.au/wps/wcm/connect/public%2Bcontent/sa%2Bhealth%2Binternet/clinical%2Bresour)
401 ces/clinical+topics/end+of+life+for+health+professionals/resuscitation+plan+7+step+pathwa
402 y+for+health+professionals)
403 4.	Dignam C, Brown M, Thompson CH. Changes in resuscitation and end-of-life
404 documentation in older patients' clinical case notes: A comparison of 2011 and 2017 practice.
405 Australas J Ageing. 2019;38:28-32.
406 5.	Fritz Z, Pitcher D, Regnard C, Spiller J, Wang M. ReSPECT is a personal emergency
407 care plan summary. BMJ. 2017;357:j2213.
408 6.	Patient Care Quality Office. Medical Orders for Scope of Treatment (MOST). Interior
409 Health Authority. British Columbia, Canada. (Accessed 18 June 2019 at
410 [www.interiorhealth.ca/YourCare/PalliativeCare/ToughDecisions/Pages/Medical-Orders-for-](http://www.interiorhealth.ca/YourCare/PalliativeCare/ToughDecisions/Pages/Medical-Orders-for-)
411 Scope-of-Treatment-(MOST).aspx)
412 7.	Hui D, De La Cruz M, Mori M, Parsons HA, Kwon JH, Torres-Vigil I et al. Concepts
413 and definitions for "supportive care," "best supportive care," "palliative care," and "hospice
414 care" in the published literature, dictionaries, and textbooks. Support Care Cancer. 415	2013;21:659-85.

416	8.	Fadel MG, Parekh K, Hayden P, Krishnan P. Improving resuscitation decisions: a

417	trust-wide initiative. BMJ Open Qual. 2018;7:e000268.

418	9.	Schmidt TA ZD, Fromme EK, Cook JN, Tolle SW. Physician orders for life-

419	sustaining treatment (POLST): lessons learned from analysis of the Oregon POLST Registry. 420	Resuscitation. 2014;85:480-5.

421 10.	Braun V, Clarke V. What can "thematic analysis" offer health and wellbeing
422 researchers? Int J Qual Stud Health Well-being. 2014;9:26152.
423 11.	Zanartu C, Matti-Orozco B. Comfort measures only: agreeing on a common definition
424 through a survey. Am J Hosp Palliat Care. 2013;30:35-9.
425 12.	Beach MC, Morrison RS. The effect of do-not-resuscitate orders on physician
426 decision-making. J Am Geriatr Soc. 2002;50:2057-61.
427 13.	Akhunbay-Fudge CY, Buss I, Ward A, Snead C, Cole M, Coulter A. Improving
428 handover of patient care using a new weekend proforma with a focus on ceiling of care. BMJ
429 Qual Improv Rep. 2014;3.
430 14.	Shermon E, Munglani L, Oram S, William L, Abel J. Reducing DNACPR complaints
431 to zero: designing and implementing a treatment escalation plan using quality improvement
432 methodology. BMJ Open Qual. 2017;6:e000011.
433 15.	Australian Commission on Safety and Quality in Health Care. National Consensus
434 Statement: essential elements for safe and high-quality end-of-life care. Sydney: ACSQHC, 435	2015.

436

# 437 Supporting Information:

##### 438 S1 Fig: Copy of 7 Step Pathway Resuscitation Plan and Alert Form

439	**S2 Text: Unabridged Case Vignettes**

Figure 1A TIF	[Click here to download Figure Fig 1A Comfort Care.TIF](https://www.editorialmanager.com/pone/download.aspx?id=24868907&amp;guid=8ce67b2b-933c-4cff-bb15-20602a89ebc4&amp;scheme=1)

Figure 1B TIF	[Click here to download Figure Fig 1B No life prolonging treatment.TIF](https://www.editorialmanager.com/pone/download.aspx?id=24868908&amp;guid=28991f7a-533a-4a2e-ac11-8020b3bd88aa&amp;scheme=1)

Figure 1C TIF	[Click here to download Figure Fig 1C -Ward measures.TIF](https://www.editorialmanager.com/pone/download.aspx?id=24868909&amp;guid=b22fa169-e822-4a37-b200-e456132b8eb4&amp;scheme=1)

Supporting Information

[Click here to access/download](https://www.editorialmanager.com/pone/download.aspx?id=24868906&amp;guid=4ffc546b-917c-4be3-bf5e-689d9ce9c4bc&amp;scheme=1)

[Supporting Information Supplementary Information.docx](https://www.editorialmanager.com/pone/download.aspx?id=24868906&amp;guid=4ffc546b-917c-4be3-bf5e-689d9ce9c4bc&amp;scheme=1)
