# Supplementary material for: The impact of language on the interpretation of resuscitation clinical care plans by doctors. A mixed methods study
Source: PLoS One. 2019 Nov 25;14(11):e0225338. doi: 10.1371/journal.pone.0225338 (PMC6876871; doi:10.1371/journal.pone.0225338)
Supplement: Attachment — Submitted filename: Response to Reviewers.docx [file pone.0225338.s004.docx]

11^th^ October 2019

Dear Drs Miller, Pitcher and Ritchie,

Thank you for your considered review of this manuscript. Given the extensive and insightful feedback received, I have presented point-by-point response to the comments raised in the table below, with references to changes made in the main text. All co-authors have reviewed the revised manuscript and approve of the amended final version for submission.

Yours Sincerely,

Colette Dignam

| **Editor Comments** | **Response** |
| --- | --- |
| 1. Ensure manuscript meets style requirements | I have reviewed the style templates to ensure the manuscript meets PLOS ONE's style requirements. In particular, I have removed the italics used to distinguish Case Vignettes and participant quotations from the manuscript text. |
| 2. Details of patient consent | Details of patient consent now included:  “Participants were informed that completion of the survey was taken for consent, in accordance with ethics approval” |
| 3. Data availability | Given your concern, I have referred this issue directly to the approving ethics committee. They have advised a restriction on uploading data to a public depository due to concerns about compromising participant privacy and confidentiality given the single-centre nature of the study. Data collection preceded recent changes to the Australian Research Council guidelines on data sharing and as such, consent for public data sharing was not sought from participants. The ethics committee has advised making ‘Data available to all interested researchers upon request’ and can be contacted directly at [Health.CALHNResearchethics@sa.gov.au](mailto:Health.CALHNResearchethics@sa.gov.au). My contact was Jasmine Kumar. |
| 4. Please ensure your work adheres to the following guideline (CHERRIES) and provide citation | I have reviewed the CHERRIES checklist and amended my methodology to reflect their reporting guidelines, including citation:  “A closed internet survey, accessed via emailed link, was sent to all doctors who had worked in general medicine at a single centre between February-August 2017. The survey was conducted between August and October 2017. The link stated the purpose, duration and nature of the survey and that completion of the survey was taken for consent, in accordance with ethics approval (HREC/17/RAH/375). Participation was anonymous, voluntary and no incentive was provided. Survey questions were pilot tested with the co-authors (MB, CHT) and physician colleagues, and refined iteratively. Questions were not randomised. The Checklist for Reporting Results of Internet E-Surveys was used as a reporting guide for this study [10].” |

| **Reviewer #1 Comments** | **Response and amendments** |
| --- | --- |
| … the title of the paper (in particular the use of the word ‘de-coding’) does not reflect the study’s findings… | I agree with this feedback. I have amended the title to:  “The impact of language on the interpretation of resuscitation clinical care plans by doctors. A mixed methods study.” |
| …The text states that 3 of the 4 authors ‘contributed equally to this work’. The nature and extent of their equal contributions are not stated…. | The online PLOS ONE submission included a page detailing the nature of contributions. Please let me know if further information is required. |
| There is a degree of irony in this paper that addresses ambiguity and misinterpretation of terminology within a document that itself has the ambiguous name ‘resuscitation plan’….. The paper could usefully discuss whether that might be resolved, at least in part, by using a different, more specific name for the plan and/or a clear statement on the form of its purpose… | Irony aside, the lack of common nomenclature is certainly an issue, particularly when attempting a thorough literature review. I have revised the opening introductory paragraph to address this:  “Clear documentation of medical treatment goals on a dedicated form helps patients receive appropriate and desired care, particularly in the setting of unexpected deterioration. Despite its recognised importance, there is no internationally accepted title for this type of documentation; terms in use include goals of care plan, resuscitation clinical care plan (resuscitation plan), and emergency treatment plan [1-3]. For the purposes of this study, we define a resuscitation plan as a document written by a doctor responsible for a patient’s care which includes a directive regarding cardiopulmonary resuscitation (CPR), but also contains recommendations and/or limitations about other interventions, such as intubation or use of life-prolonging antibiotics. Documentation should record the goals of care following a discussion with the patient or substitute decision-maker (SDM) about the treatment preferences, informed by the doctor’s medical assessment of likely treatment outcomes [1,2].” |
| A weakness of this study, which should be acknowledged in the text, is that it has asked doctors to interpret (in the context of case vignettes) free-text phrases used on resuscitation plans from 2017 that were examined in a previous study…..the current paper fails to acknowledge the crucial role of education (including audit, appraisal and feedback) in persuading doctors to record clear recommendations and stop using ambiguous phrases. If these phrases are in widespread use, that appears to indicate failure of adequate staff education in the completion of the 7-step form | On a practical level, the data collection for this study began 4 months after the initial study data was collated, based on preliminary analysis of the first study’s findings. The aim was to demonstrate that undefined language IS affecting care in the first instance (which, until now, was a theoretical concern). Designing and carrying out an educational campaign in this time frame was not possible. I have acknowledged this in limitations.  “In addition to on-site education at the time of its launch at each new site, training about the 7-Step Pathway is available through online educational modules. The adequacy of these interventions to support the completion and interpretation of the form is a confounding factor that has not been addressed in this study. The capacity for a renewed education strategy and dedicated resources to improve the process, as opposed to changing the form, remains an area for future research.” |
| In more than one place, the text states ‘Terminology was neither consistently interpreted nor applied…’. I think that the authors intended to state that terminology was neither interpreted consistently nor applied consistently, not that the ‘terminology’ was not applied. | The wording has been changed throughout the paper to reflect this clearer suggested phrasing. |
| The paper refers to ‘not for Cardiopulmonary Resuscitation orders’ but does not make clear the legal status of such documents in Australia. | The introductory text has been changed to reflect the relationship between legally binding Advance Care Directives and the 7-Step Pathway:  “In 2014, the 7-Step Pathway Resuscitation Plan and Alert form (7-Step form) (S1 Fig 1) was introduced as an integral part of implementing the *Advance Care Directives Act* *2013* (SA) to provide a clinical tool to reinforce the principle of self-determination; the individual’s right to refuse treatment including CPR.”  “…While the 7-Step form is not legally binding in isolation, it may be used to reflect previously documented binding treatment refusals that a patient has recorded on an Advance Care Directive. ^“^ |
| The paper states that the 7-step form is being used in all ‘public tertiary hospitals’ in South Australia…This terminology is potentially ambiguous for an international readership, so it would be helpful for the text to define the type, size and number of hospitals covered by this term. | See next response |
| It would be helpful and of great interest also to understand what arrangements are in place to communicate recommendations for the emergency care and treatment of patients who have not been admitted to this type of hospital, or patients transferred or discharged from a tertiary hospital. | “This resuscitation plan is now used in all six South Australian urban public referral hospitals and is available in smaller rural hospitals and clinics staffed by primary care providers, it is also recognised as a treatment guide by ambulance officers [3]… The plan should be communicated to the primary care provider on hospital discharge, and a carbon-copy provided to the patient.” |
| The paper implies in a single sentence that the 7-step form is very similar to 'international counterparts'. This is not strictly true. There are substantial differences, and it would be appropriate to identify these in the text and to consider them in subsequent discussion of possible options for improving the design of the 7-step form to overcome the problems identified in this study. | I have added the following to Introduction:    “In line with this study’s definition, international examples of resuscitation plans also contain a CPR directive, a description of treatment limitations or recommendations, and a record of patient/SDM involvement. Key differences between examples include the relative focus on free text documentation vs standardised tick box care options, whether the document is used for a specified length of time or indefinitely (as is the case with the 7-Step form), and the involvement of senior staff in creating a plan [1,5,6].” |
| There is published evidence that using a discussion process and form that focus on and record treatments that would be wanted and should be considered to help to achieve the person’s goals of care, not primarily on treatment to be withheld, are preferred by both patients and by healthcare professionals. The ‘7-step form’ appears to focus more on withholding treatment rather than on giving realistic care and treatment to meet the person’s goals of care. As such, it does not appear truly person-centred or evidence-based. | The government-endorsed evidence-based approach that underpins form completion is perhaps under-represented in the manuscript. I have added the following to Introduction:  “The form and underlying Resuscitation Planning Policy Framework were developed to address previously identified inadequacies in the process of resuscitation decisions and documentation, with a renewed emphasis on transparent patient-doctor discussion that would result in desired and appropriate medical care, while protecting the right to withhold futile interventions.    The completed form represents the final step in a process of creating a care plan in conjunction with the patient; a stepwise approach that is locally known as the 7-Step Pathway. In accordance with current evidence base, the 7-Step Pathway includes medical assessment, open consultation with the patient about their treatment goals, and a transparent discussion of appropriate treatment within the framework of known co-morbidity and quality of life [3,4]. ” |
| In the published report of their previous study (reference 4) the authors concluded that the current Alert Form (‘7-step form’) could be improved. The tick-box list in section 4 of the form focuses firstly on emergency treatments to be withheld, emphasised using red print. Treatments that ‘will be provided’ are relegated to a rather less prominent free-text box below. Even the wording in this box is ambiguous. This box should list treatments that would be wanted and should be considered – and provided if they are needed. I would like to see much more discussion about the substantial potential to improve the form and to try to ensure that the form is used to support good communication, good medical practice and person-centred care, rather than primarily supporting the healthcare system. | I have addressed this and the following comment with a further paragraph in the Discussion;  An ideal resuscitation plan therefore needs to strike a balance between patient advocacy, a clear medical handover, and the unpredictable nature of future hospital admissions. While junior doctors in this study were in favour of increased standardised choices such as ‘Not for non-invasive ventilation’, a list of this nature will inevitably be incomplete and maintains a focus on the treatment that will be withheld. Contemporary alternatives include the model exemplified in the USA-based POLST (physician’s orders for life sustaining treatment) model, where patients are divided into three broad treatment approaches; full, limited and palliative. The increased clarity and ease of use in this three-tiered care model potentially comes at the expense of a nuanced and individualised patient-centred approach. The ReSPECT (Recommended Summary Plan for Emergency Care and Treatment) model in United Kingdom provides ample scope for individualisation and patient involvement in the form of visual scales balancing curative and symptom-based care. This form requires the doctor to accurately document treatment limitations in free-text format however, thereby risking the use of undefined or ambiguous terminology that proved so problematic in this study. |
| …. There are several potential ways in which the form could be modified to make it more person-centred and less likely to lead to varying interpretation and incorrect decision making in an emergency, and I would like to see the discussion expanded to consider these other options and the advantages and disadvantages of each. Trying to create a comprehensive list of tick-box treatment options to cater for every person's individual needs seems likely to make the form unduly long and cumbersome for all, and trying to limit the number of treatment options will inevitably fail to cater for all individual needs…. | As above |

| **Reviewer #2** | **Responses** |
| --- | --- |
| You mention in results in the abstract that you had clinical significant differences in treatment and I do not think it has been addressed as to why you think that was. Why were more senior faculty more likely to be aggressive in treatment? Training differences, generational differences? | I have expanded on this in the discussion:  “Interns, who are inexperienced in clinical decision-making and are used to following senior orders, were also more likely to both closely follow a resuscitation plan and consider it a useful decision-making tool. This may in part explain the statistical differences in treatment approaches seen in the case vignettes. Internationally, many resuscitation plans require a secondary signature from a senior officer, which may be one practical solution to improve cohesion in the approaches of junior and senior staff. |
| Grammatical and sentences structure suggestions | The suggested amendments have been adopted throughout the paper. |
|  |  |
